# Phenylboronic acid in targeted cancer therapy and diagnosis

**DOI:** 10.7150/thno.104558

**Published:** 2025-03-03

**Authors:** Mila Radan, Ivana Carev, Mladen Miloš, Marina Tranfić Bakić

**Affiliations:** Faculty of Chemistry and Technology, University of Split, Ruđera Boškovića 35, 21000 Split, Croatia.

**Keywords:** phenylboronic acid, cancer, drug delivery systems, sialic acid, antitumor activity

## Abstract

Over the past few decades, phenylboronic acid (PBA) and its derivatives have gained attention for their biological activity. More recently, they have gained interest for their application in targeted cancer therapy, owing to their ability to selectively and reversibly bind to sialic acids on the cell surface through the formation of boron ester bonds. Initially, the research was focused on the antitumor properties of simple PBA derivatives. However, it has since expanded to include more complex nanomaterials and drug-delivery systems that exploit the unique properties of PBA for enhanced therapeutic efficacy. This paper presents a comprehensive review of the physico-chemical background of PBA-based drugs in cancer therapy, provides insight into recent advancements in PBA-based systems for targeted drug delivery and their role in improving antitumor efficacy, and offers a perspective on future research and development in the field.

## 1. Introduction

While cancer therapies and diagnostics have undergone revolutionary advancements in the past century, the need for continual improvement remains one of the greatest challenges in medicine today. The concept of "magic bullets" - agents designed to selectively attack cancer cells while at the same time sparing healthy tissues, introduced by the Nobel Laureate Paul Ehrlich, has laid the foundation for modern chemotherapy 1. Even though numerous advancements in molecular biology, medicinal chemistry, and genetics have been made, the success rate for bringing new cancer drugs to clinical use remains only approximately 5% 2, primarily due to inadequate efficacy and toxicity issues often identified during late-stage clinical trials. Integration of biomarkers into preclinical drug discovery could address these issues and enhance the selection of promising compounds for clinical evaluations. Additionally, contemporary research aims at developing multi-targeted therapies to tackle the complexity of cancer, alongside personalized medicine strategies tailored to individual patients' needs.

In line with these demands, phenylboronic acid (PBA) and its derivatives have recently emerged as promising agents capable of targeting cancer cells and provoking specific biological responses, revealing an unprecedented potential in cancer diagnostics and therapy 3,4. Understanding the structural interactions of these compounds with cancer-specific biomarkers can enable the design of improved targeted therapies that minimize off-target effects and improve treatment efficacy. This approach facilitates the development of novel diagnostic tools and therapeutic agents that exploit unique cancer cell surface features. Additionally, nanocarriers such as liposomes and micelles incorporating PBA could provide a more personalized approach to cancer therapies, focusing on mechanisms of passive and active targeting for tumor treatment, potentially leading to more effective and less toxic cancer treatments.

**Figure 1:**
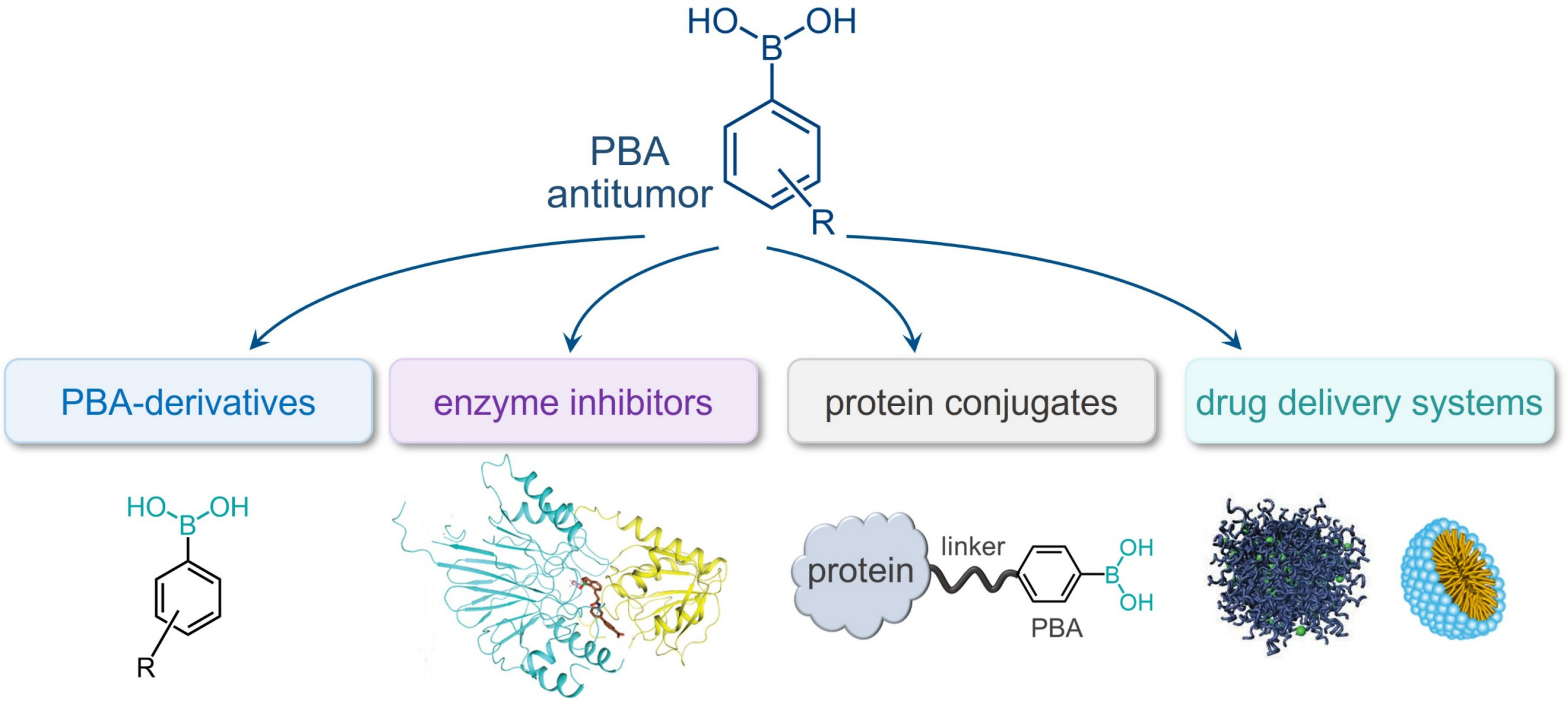
Types of PBA-based systems for targeted cancer therapy and diagnosis.

**Figure 2:**
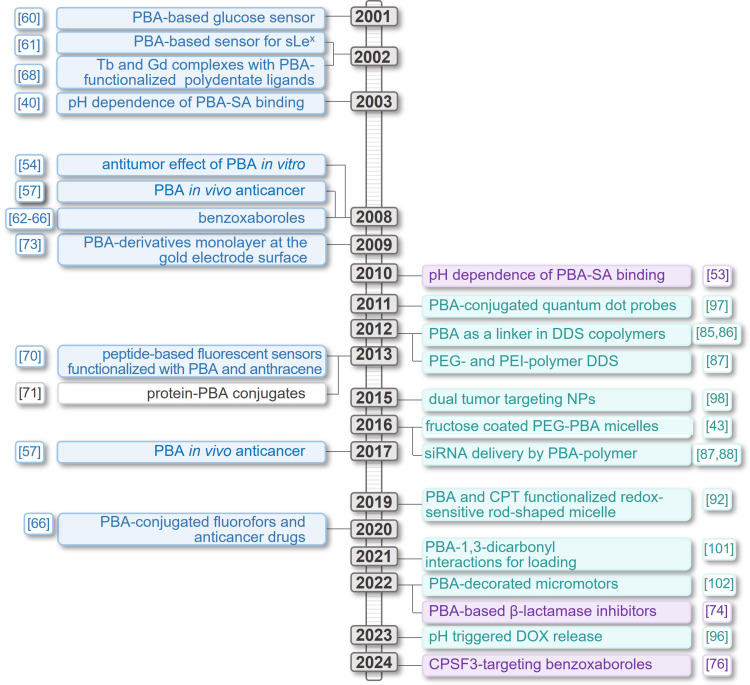
Timeline presenting significant advancements made in PBA-based systems for targeted cancer therapy and diagnosis.

This review thoroughly explores the chemistry of PBA in relation to the specific morphology of cancer cells and different types of PBA-based anticancer systems (Figure 1). It provides insight into the evolution of the field from simple PBA-derivatives acting as sensors to highly complex drug delivery systems designed to overcome the challenges of tumor treatments (Figure 2).

## 2. Chemical basis of the cancer-targeting activity of phenylboronic acid

**Figure 3:**
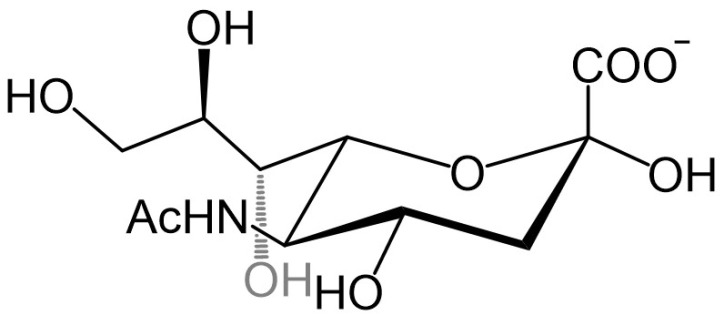
The structure of 5-N-acetylneuraminic acid, Neu5Ac.

The surfaces of cancer cells exhibit distinct chemical and morphological properties compared to healthy cells, largely due to alterations in their membrane structure. These changes impact cellular function, including cell-cell interactions, apoptosis, immune response, and tumor progression 5-11. Among other membrane components, glycolipids and glycoproteins on the cell surface represent the first contact of the cell with its surroundings. They are involved in various biological processes such as cellular communication, molecular recognition, and the reception of external signals 10. Several recent studies showed that many glycans on the surfaces of tumor cells are excessively expressed compared to healthy cells, especially sialic acids (SAs) which stand out due to their special location at the terminal end of glycans in glycolipids and glycoproteins. Sialic acids are anionic monosaccharides with *N*- and *O*-substituted derivatives of neuraminic acid and in humans, the dominant one is the 5-*N*-acetylneuraminic acid (Neu5Ac, Figure 3).

Cancer malignancy and metastasis are often associated with hypersialylation 12-15 and this structural feature of tumor cells can be exploited for the development of new diagnostic and therapeutic tools in which the glycans serve as biomarkers for pathological states and targets for cancer diagnosis and antitumor therapeutics 16-22.

Lectins are compounds in biological systems that exhibit selective binding to sugars of cell-surface glycans. However, their use as potential antitumor agents is impeded by several disadvantages owing to their protein nature: immunogenicity, inflammatory action, cellular toxicity, and mitogenic stimulation, as well as time-consuming and generally expensive large-scale production, relatively low chemical stability, and problematic long-term storage 23. As an alternative to lectins, a number of smaller non-protein lectin-mimics compounds that overcame the shortcomings of lectins have also demonstrated reversible binding to glycans and have therefore recently attracted much attention, especially compounds based on PBA 24-27.

**Figure 4:**
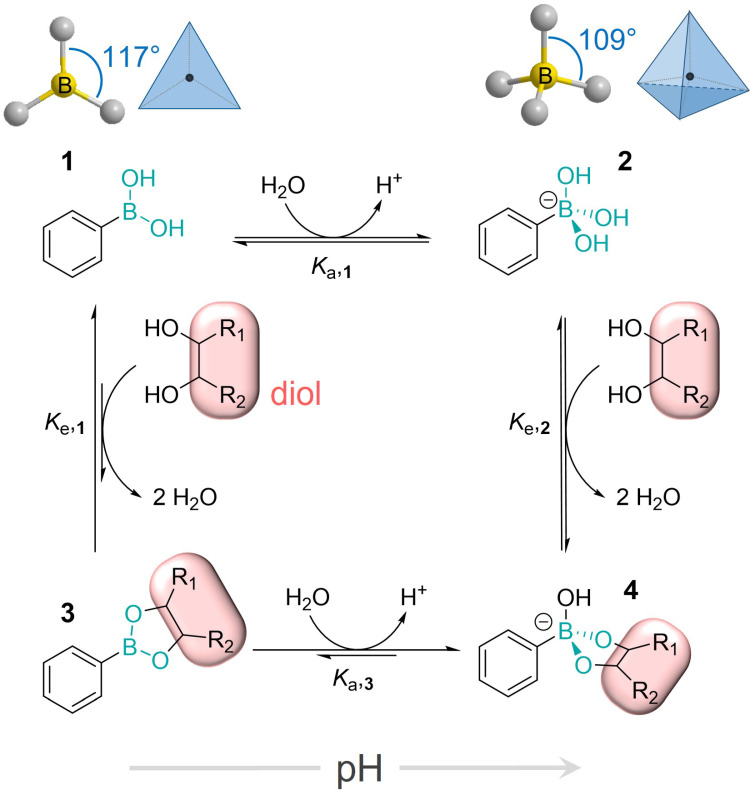
The reaction network of the pH-dependent formation of the boron ester which includes the interconversion of hybridization and geometry of the boron atom.

In aqueous solutions at physiological pH values PBA effectively interacts with glycans and binds to 1,2- and 1,3-diol groups by forming five- and six-membered cyclic esters, respectively, as shown in Figure 4. Interestingly, even though this binding occurs through the formation of covalent boronate ester bonds, it is reversible, as well as significantly pH-dependent 4,28-31. This is the result of the reaction network comprising the two protonation and two esterification equilibria. In general, the boron atom in its uncharged form has sp^2^-hybridization and forms trigonal planar molecules through the formation of 3 covalent bonds, leaving one of its sp^2^-orbitals empty. This makes boron compounds Lewis acids, readily available for a reaction with a nucleophile. In this reaction, the two nucleophilic electrons are brought to the boron atom resulting in its negative charge and interconversion to sp^3^-hybridization state with tetrahedral geometry 32-35. The hybridization and geometry interconversion are observed also in the acid-base reaction of PBA with the p*K*_a,**1**_ of 8.8, where the planar uncharged form **1** upon nucleophilic reaction with a hydroxy group converts to the tetrahedral and negatively charged form **2** 36,37**.**

Both of these forms can be subject to esterification by diols and the corresponding esterification reactions are equilibrium processes resulting in the corresponding boronate ester (**3**, **4**) as well as the free PBA forms (**1**, **2**) being present in solution at all times 38. On the other hand, the acid-base equilibrium of the resulting boronate ester **3** (described by the acidity constant *K*_a,**3**_) is significantly shifted towards the negatively charged species **4** 39. Just as the PBA *K*_a_ values are 2 to 4 orders of magnitude higher than the *K*_a_ of its esters with numerous monosaccharides, the esterification constants for the neutral PBA are 2 to 4 orders of magnitude lower with respect to the negatively charged basic species.

Considering the fine interplay of the four equilibria in the described reaction network, it is not surprising that the overall stability of boronate esters is influenced by several factors: the acidity constants *K*_a,**1**_ and *K*_a,**3**_, the esterification constants *K*_e,**1**_ and *K*_e,**2**_, pH, and the diol *K*_a_. At low pH PBA exhibits low affinity towards diols, but this affinity increases significantly between pH 4 and 7. In more basic conditions, however, the esterification constants decrease as pH increases. This suggests that the strongest binding of PBA and diols can be achieved at pH ~ 7, but the precise value of the pH at which the binding is the strongest is to a certain extent dependent on the p*K*_a_ of the diol 38.

**Figure 5:**
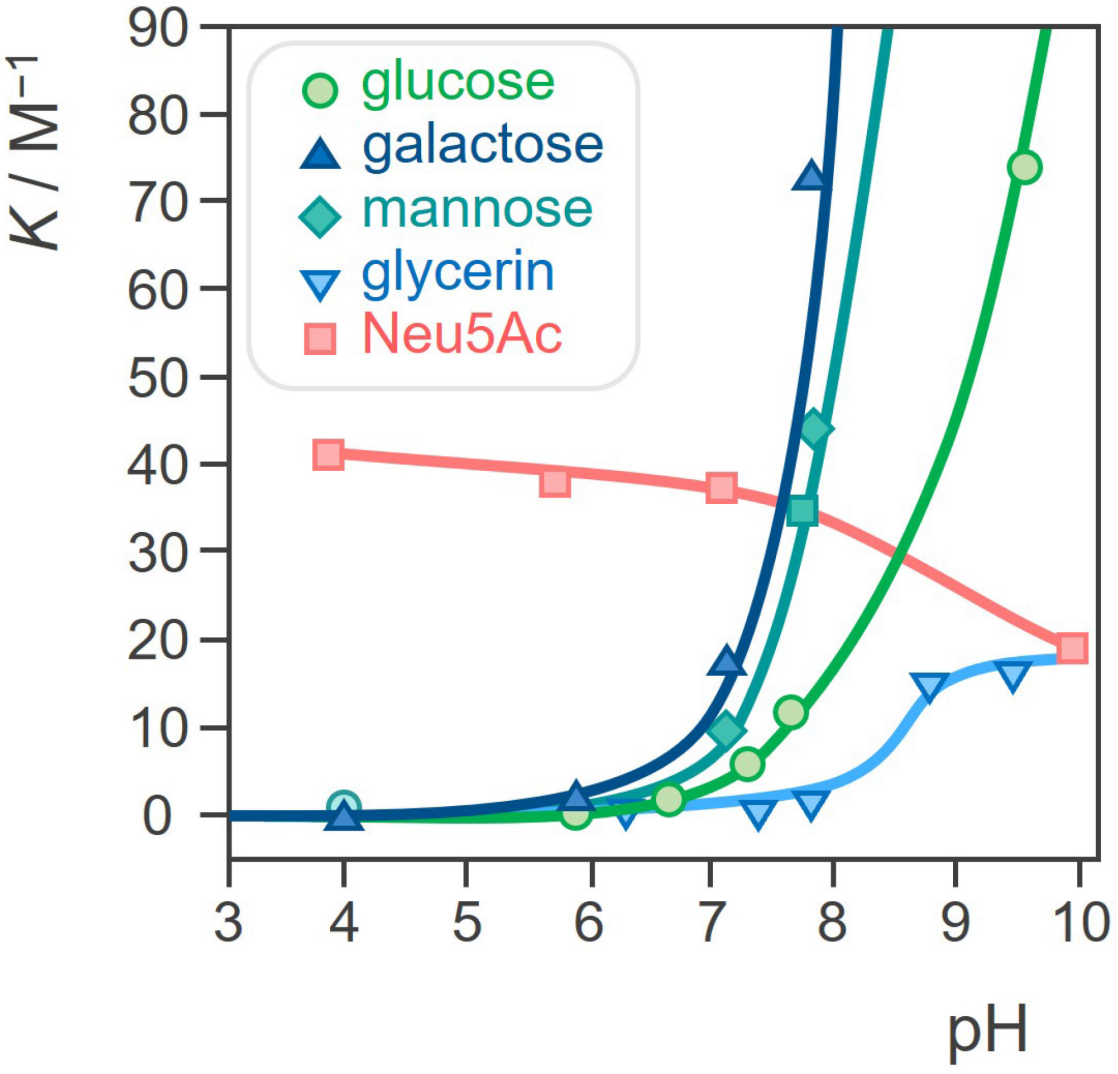
pH dependence of the binding constants for the esterification of a PBA derivative with different diols, namely some common monosaccharides and glycerol. Adapted with permission from 39, copyright 1959 the American Chemical Society.

In 2003 Kataoka *et al.* found that the profile of the dependence of the binding strength of PBA and SA on pH significantly differs from the profile for other monosaccharides 40. More precisely, the binding affinity of PBA towards SA is significantly higher, about 40 times compared to other sugars. Even more, unlike most other saccharides which are the most stable around neutral pH, the stability of the ester formed between PBA and SA increases as the pH decreases (Figure 5). Later, these findings have been further confirmed through several broader studies 40-46. As a result of this peculiar characteristic, PBA will have higher affinity for the SA on the surface of normal healthy cells in a physiological environment with pH 7.4, while in tumor regions where pH is 6.5 the specific binding will be stronger. In other words, PBA can recognize the overly expressed SA of the tumor cells. This pH sensitivity is particularly advantageous in the context of cancer treatment, since the acidic microenvironment of tumors enhances the selective binding of PBA to cancer cells, facilitating targeted drug activity.

This unusual and highly specific behavior of PBA allows its application as a diol ligand in various contexts, including the development of glucose sensors, use in chromatographic separation of carbohydrates, as protecting groups for diols and amines, and in drug delivery 32,47. Even more, these properties make PBA a suitable and extremely promising platform for innovative research for the development of systems for rapid detection of disease-related cell-surface glycoproteins and targeted drug delivery. In addition, the selective binding of biologically active PBA-decorated compounds preferably on the surface of tumor cells ensures their direct exposure to the therapeutic and thus a more efficient biological effect 48-53.

As the first step towards medical use, Plopper *et al.* studied the antitumor effect of PBA *in vitro* on human prostate and breast cancer lines, comparing it to boric acid 54,55. They showed that PBA even at micromolar concentrations acted as a selective inhibitor of cell migration and decreased viability, while the same effect was not observed for nontumorigenic cells. This suggested the potential of PBA as anti-metastatic and anti-proliferative agent.

Moving forward, in 2017, Miloš *et al.* performed the first *in vivo* study of the antitumor effects of PBA on mouse mammary adenocarcinoma and squamous carcinoma cells and proved unsubstituted PBA to be highly cytotoxic for several types of tumor cells in mice 56. Furthermore, three routes of application were considered, and intraperitoneal administration gave the best results, with good toleration by mice, even after repeated large-dose administrations.

Compared to unmodified PBA, the development of PBA derivatives has been driven by the need for improved binding affinity, selectivity, and stability under physiological conditions. These derivatives are often functionalized with electron-withdrawing groups or other modifications to enhance their selective interactions with diols, ensuring better targeting in complex biological environments, which is crucial for increasing the efficacy of targeted therapies and reducing off-target effects 45. During the last few decades, PBA derivatives have found numerous applications and became the center of interest of modern research in chemistry and related interdisciplinary sciences, biomedicine, analytical sciences, and functional materials 31,57-59.

## 3. Derivatives of phenylboronic acid in cancer diagnostics and therapeutic interventions

**Figure 6:**
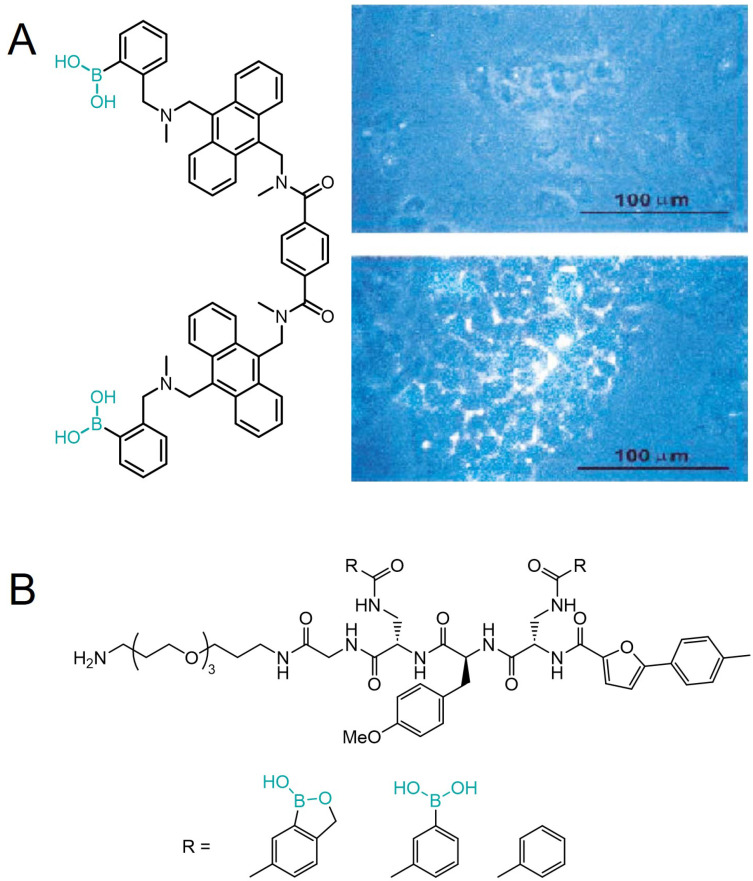
Structures of A) a fluorescent sLe^X^ sensor by Wang *et al.* and its use in fluorescent labeling of sLex-expressing cells (bottom) and non-expressing cells (top), and B) a benzoxaborole, PBA, and phenyl substituted TF antigen sensors by Hall *et al.* Adapted with permission from 61, copyright 2002 Elsevier.

Although various boronic acids have been known for more than a hundred years, it wasn't until the beginning of this century that they started to attract more interest which led to the emergence of new areas of discovery. Firstly, the research revolved mostly around employing the specific properties of PBA for the development of new, efficient, highly sensitive, and selective sensors for saccharides, primarily glucose due to its biochemical importance 60. Shinkai *et al.* developed a series of PBA-based glucose sensors 3,4, and later continued to expand their studies to more complex saccharides. In 2002, Yang and coworkers described a fluorescent sensor for sialyl-Lewis X (sLe^x^), a cell surface tetrasaccharide containing SA that plays an important role in cancers and metastasis. The sensor incorporated two PBA moieties for selective binding to the disaccharide and two anthracene moieties to induce a fluorescence response upon binding (Figure 6A) 61. In the same paper, they demonstrated the efficacy of this sensor in selective labeling of the tumor cells expressing sLe^X^ for fluorescence imaging, while the non-sLe^X^-expressing cells remained unstained, making this receptor the first synthetic selective cancer diagnostic tool of this type.

A few years later, Hall and coworkers found that benzoxaborole, a cyclic monoester of 2-(hydroxymethyl)phenylboronic acid, unlike PBAs, binds nonreducing saccharides, namely glycopyranosides, in neutral water 62,63. Soon after, they continued to develop a library of benzoxaborole- and PBA-based receptors for Thomsen-Friedenreich (TF) antigen, a Gal-β-1,3-GalNAc disaccharide which is a known tumor marker expressed in 9 out of 10 cancers 64. As diboronic acids have a higher affinity for saccharides than simple PBAs 65, they applied the same concept and attached the two boron-containing substituents to the central aminoacids of a polypeptide decorated on one end with a glycol moiety to increase the compounds' solubility (Figure 6B). Utilizing a modular approach, they created a library of receptors that effectively recognize carbohydrates in an aqueous environment. These receptors leverage the unique properties of benzoboroxoles to form reversible complexes with hexopyranosides, enhancing binding affinity through multiple interaction modes, including boronate formation, hydrogen bonding, and hydrophobic interactions. Among them, one receptor showed strong selectivity for the TF-antigen clearly demonstrating the superiority of the bisbenzoxaborole moiety over the boronic acid moiety regarding selective high-affinity binding to pyranoses 23. It is quite surprising that despite these very promising results, the receptors were never further investigated for their biological activity and especially for their potential antitumor properties.

In addition, some benzoboroxole-derived compounds have been shown to exhibit antiproliferative activity. For example, the Suman group synthesized a series of *N*-benzoboroxolylureas and *N*-nitrosoaminobenzoboroxoles derived from aminobenzoboroxoles, several of which showed promise in preliminary cytotoxicity evaluations against human breast cancer and pancreatic cancer cell lines 66.

As new insights into the specific biochemical behavior of PBAs emerged in the late 1990s and early 2000s, considerable efforts have been made to exploit their biological activity, and extensive research is currently underway to investigate their potential for medical applications. In the study by Zhang and colleagues in 2020, PBA derivatives were conjugated with dicyanomethylene-4H-pyran fluorophores as well as with the anticancer drug SN-38 67. The cellular uptake of PBA-dye conjugate by hepatocellular and lung carcinoma cells was significantly higher than non-PBA-containing counterparts, and PBA-SN-38 conjugates achieved up to 3- to 7-fold increases compared to the cytostatic drug irinotecan. Despite improved delivery, the drug-PBA system did not enhance the anti-proliferative effect against liver trumor cell lines, likely due to the stability of the system and consequent drug inactivation.

The Sporzyński group evaluated the antiproliferative action of a series of PBA and benzoxaborole derivatives for several cancer lines 68. They noted that the substituent type and position clearly affected the antiproliferative activity of the compound as well as its p*K*_a_. The two most active derivatives were identified and used to further study cell cycle arrest and apoptosis induction in ovarian cancer cells. The research further demonstrated that these compounds significantly increase the percentage of cells in the G2/M phase and promote the formation of tetraploid cells, which is a hallmark of mitotic catastrophe. This cell cycle arrest was accompanied by a marked increase in p21 protein levels, which is known to inhibit cyclin-dependent kinases (CDKs), thus preventing cell cycle progression. Interestingly, p21 accumulation was not associated with β-tubulin degradation, indicating that the mechanism of action is distinct from traditional microtubule-targeting agents. Instead, the G2/M arrest resulted in the failure of proper mitotic division, ultimately leading to apoptosis *via* caspase-3 activation. This phase cycle-specific induction of apoptosis requires further studies to clarify their mechanisms of action and therapeutic potential in cancer treatment.

In the context of PBA derivatives known for targeting SAs on cancer cells, terbium and gadolinium complexes with polydentate ligands functionalized with PBA have also been examined for their tumor-targeting abilities 69,70. These compounds demonstrated superior effectiveness by combining the covalent binding with SA and electrostatic interaction of the positively charged complex with negatively charged cell surfaces. This dual interaction approach highlights the potential of PBA-functionalized lanthanide complexes for molecular imaging and radiotherapy applications, furthering the use of PBA derivatives in cancer-targeted diagnostics and treatment.

In 2013 Xu *et al.* presented five peptide-based fluorescent sensors functionalized with PBA and anthracene moiety responsible for fluorescent response, varying in the peptide backbone 71. These water-soluble and biocompatible compounds were designed for *in situ* recognition of and targeted fluorescent imaging of cancer cells. Among them, one compound was shown to highly specifically recognize the sLe^x^ cell-surface glycan and fluorescently label the targeted cells. The selective recognition of cancer cells is the result of the specific recognition between the specific tripeptide sequence of the sensor and integrins associated with cancer.

A more complex PBA-based system used in cancer diagnosis and therapy was described by Miyahara and coworkers 72,73. It consisted of a monolayer of PBA derivatives self-assembled at the surface of a gold electrode which was used as a noninvasive potentiometric sensing system for selective detection of tumor metastasis cells by recognizing their surface SA. Even more, this system showed the ability to quantify the degree of metastasis in mice cell suspensions.

Martinelli 74 and Martinelli 75 focused on the solid-phase synthesis and evaluation of PBA-based tumor-targeting MRI (magnetic resonance imaging) contrast agents, with the latter reporting enhanced performance of dimeric analogues. Their study introduced a novel approach to synthesizing paramagnetic macrocycles functionalized with phenylboronic moieties, which hold promise for MRI applications due to their cancer cells targeting ability and local contrast enhancement. The key innovation lies in the solid-phase synthesis strategy employed, which enabled the successful preparation of DOTA-EN-PBA and DOTA-EN-F2PBA ligands (DOTA = 1,4,7,10-tetraazacyclo-dodecane-1,4,7,10-tetraacetic acid, EN = ethylenediamine). Notably, the latter incorporates fluorine substituents to increase the acidity of the boronic moiety, further enhancing its tumor-targeting capabilities. This research represents a significant advancement in the development of MRI contrast agents for precise cancer imaging, offering potential benefits for early detection and diagnosis.

Ultimately, one PBA derivative, 4-boronophenylalanine, is among the agents currently used in clinical trials for boron neutron capture therapy (BNCT). This is a type of radiotherapy used for invasive and localized cancers where first a boron-containing (isotope ^10^B) and tumor-localizing drug is administered, and then the patient is radiated with epithermal neutrons. Boron atom has a high potential for capturing the neutrons which results in its isotopic decay releasing “high-energy α-particles that kill the cancer cells that have taken up enough ^10^B”. Currently, the request for new boron-delivering agents that would have lower systemic toxicity and achieve higher concentrations in tumor tissues has led to the development of different systems such as boron-containing nanoparticles (NPs), boronated polyamines, etc. 76.

## 4. Derivatives of PBA as enzyme inhibitors

PBA derivatives have emerged as potent agents in biomedical research due to their ability to form covalent bonds with biological molecules. This property also enables enzyme inhibition by PBA and of interest is their anticancer potential through their role as proteasome inhibitors. Trippier *et al.* highlighted the success of bortezomib, a boronic acid-based proteasome inhibitor, which inhibits cancer cell growth by targeting proteasome-mediated protein degradation 49. Bortezomib's mode of action involves forming covalent bonds with the proteasome, leading to the inhibition of protein degradation, cell cycle arrest, and apoptosis. The success of bortezomib has inspired the development of PBA derivatives, including boronic-chalcone analogs that show strong activity against breast cancer cell lines by inhibiting tubulin polymerization, further underscoring the therapeutic versatility of PBA compounds. This trend toward proteasome targeting continues to be a key focus in cancer therapies, given the crucial role of proteasomes in tumor progression.

Another promising application of PBA derivatives lies in their role as β-lactamase inhibitors, as demonstrated by Zhou *et al.* 77. Triazole-substituted PBA derivatives were shown to inhibit KPC-2 β-lactamase, an enzyme responsible for antibiotic resistance. The structure-activity relationship (SAR) studies revealed that the introduction of triazole moieties enhanced enzyme binding affinity and stability. Even more, Xu *et al.* introduced a series of indolylarylsulfones bearing PBA functionalities as potent HIV-1 non-nucleoside reverse transcriptase inhibitors with remarkable activity against wild-type HIV-1 and several resistant strains 78.

Although the primary focus of these studies was on antibacterial and antiviral activity of PBA derivatives, the findings emphasize their structural versatility and highlight their broader potential in targeting critical enzymes involved in disease pathways. This ability to fine-tune the p*K*_a_ and binding properties makes PBA derivatives attractive candidates for diverse therapeutic applications, including anticancer therapies where enzyme inhibition plays a crucial role.

An interesting study by the Plopper group showed that PBA acts as an inhibitor of Rho family of GTP-binding proteins RhoA, Rac1, and Cdc42 in metastatic prostate cancer cells cells but not in normal prostate cells 54,55, which also impacts the downstream targets of these proteins. Furthermore, PBA decreased the activity of ROCKII as well as phosphorylation of myosin light chain kinase. Overall, cancer cell growth was arrested and cell migration was inhibited in rapidly proliferating cells.

In addition, a series of benzoxaboroles has been found to induce cancer cell death by targeting CPSF3, an enzyme essential for mRNA processing and transcription termination 79. These compounds bind to the active site of CPSF3 and act as inhibitors, leading to widespread gene downregulation and eventual cell death.

## 5. Protein conjugates of PBA

**Figure 7:**
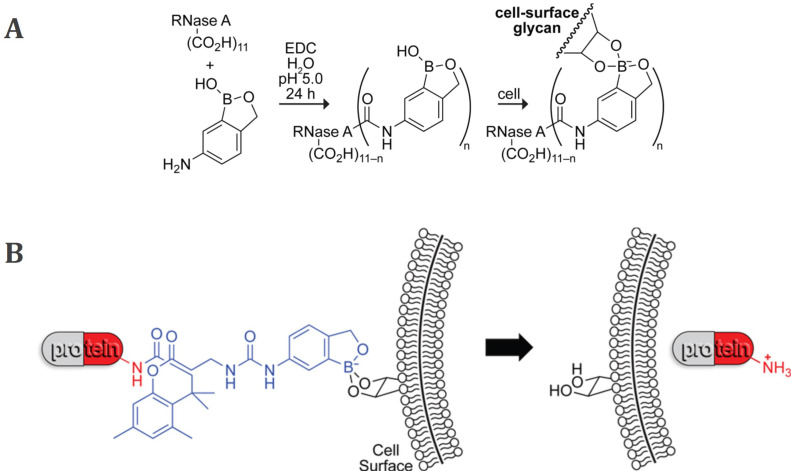
A) Boronation of RNase A *via* amide bond and B) protein boronation strategy through an immolative linker (blue) and binding of the conjugates to cell surface glycans followed by internalization of the native protein by Raines *et al.* Adapted with permission from 80,82, copyright 2012 and 2016 American Chemical Society.

As previously mentioned, the development of new approaches for tumor targeting was linked to research on synthetic lectin mimetics. In this regard, the early attempts to achieve an antitumor effect often involved protein-PBA conjugates, expected to have biological activity similar to lectins. The first example of such bioconjugates was a benzoxaborole linked to a RNase A by an amide bond reported by Raines *et al.* in 2012 (Figure 7A) 80. It was shown that the protein affinity for the human erythroleukemia cells increased upon boronation, as well as its cellular uptake. Even more, boronated RNase A inhibited the human erythroleukemia cells proliferation. The recognized potential of boronates as effective lectin mimetics was further exploited in a similarly designed conjugate of the bovine serum albumin (BSA) with PBA 27,81. The conjugate was deposited on silica beads as well as on a gold chip, and in both cases, efficient binding of sugars and SA on their surface was demonstrated. Although the approach of binding proteins directly to boronic acids *via* amide bond promotes protein binding to cancer cells and its internalization, unfortunately, the catalytic activity of boronated proteins is significantly reduced compared to their native form 80.

In 2016, Raines *et al.* proposed an improved strategy using a benzoxaborole-based carrier that binds the proteins through an immolative linker (Figure 7B) 82. In such a conjugate, the covalent modification of the amino groups of the protein is bio-reversible, since esterases present inside the cell, but not outside, can cleave the bond between the linker and the protein, releasing the native protein into the cell cytosol. Such boronation of the protein chemically inactivates it making it less cytotoxic in the extracellular fluid, and at the same time facilitating its cellular uptake and internalization. Overall, the RNase A conjugate showed a decrease in the IC_50_ value compared to nonconjugated RNase A of human myelogenous leukemia cells and inhibition of their proliferation.

## 6. Nanoscale drug delivery systems for cancer therapy based on PBA derivatives

Around the time PBA derivatives were shown to selectively recognize SA, liposomes, micelles, microspheres, and other nanostructures started to gain attention as carriers for diagnostic agents and therapeutics. The use of such drug carriers in biomedicine has several significant advantages compared to the conventional drugs: i) they are biocompatible and enable the transport of poorly soluble compounds to the targeted tissues, ii) they can alter the pharmacokinetics of the drug by ensuring its longer circulation due to the protection from immune system degradation, thus allowing for prolonged release which reduces the overall drug dose needed and minimizes systemic toxicity, iii) they can be designed to deliver drugs specifically to the desired site, increasing bioavailability and concentration in pathological areas, iv) they reduce and even eliminate the unwanted side-effects, v) they can be engineered for controlled drug release over time 83,84. These beneficial effects combine to significantly enhance the efficiency of the drug. Consequently, such formulations have become increasingly widespread, with the most notable example being COVID-19 mRNA vaccines, where lipid NPs serve as the drug delivery system 85. Significant efforts have been devoted to developing new formulations, leading to remarkable advances in the field.

### 6.1. PBA as linker in DDS copolymers

Interestingly, the first use of PBA in drug delivery formulations was not for tumor targeting, but rather as a crosslinker between the polyethylene glycol (PEG) and cholic acids blocks, which self-assemble into micellar copolymers called telodendrimers and thereby increasing their stability 86. The resulting micelles were loaded with paclitaxel (PAX), a widely used chemotherapeutic drug, and it was shown that the drug release was slow at physiological pH of 7.4, but lower pH triggered the release of PAX enhancing the inhibition of growth of ovarian cancer cells.

A more recent example employing a similar approach was described by Shuai and coworkers 87 who developed another pH-sensitive polymeric drug carrier composed of a hydrophobic and hydrophilic polymer linked by phenylboronic ester, forming an amphiphilic structure that self-assembled into stable PTX-loaded NPs with high encapsulation efficiency. These NPs have excellent drug encapsulation and pH-triggered drug-release capabilities, leading to low systemic toxicity, but high anticancer activity and improved pharmacokinetics compared to free PAX, both *in vitro* and *in vivo*.

### 6.2. PEG- and PEI-based DDS with PBA as the recognition unit

**Figure 8:**
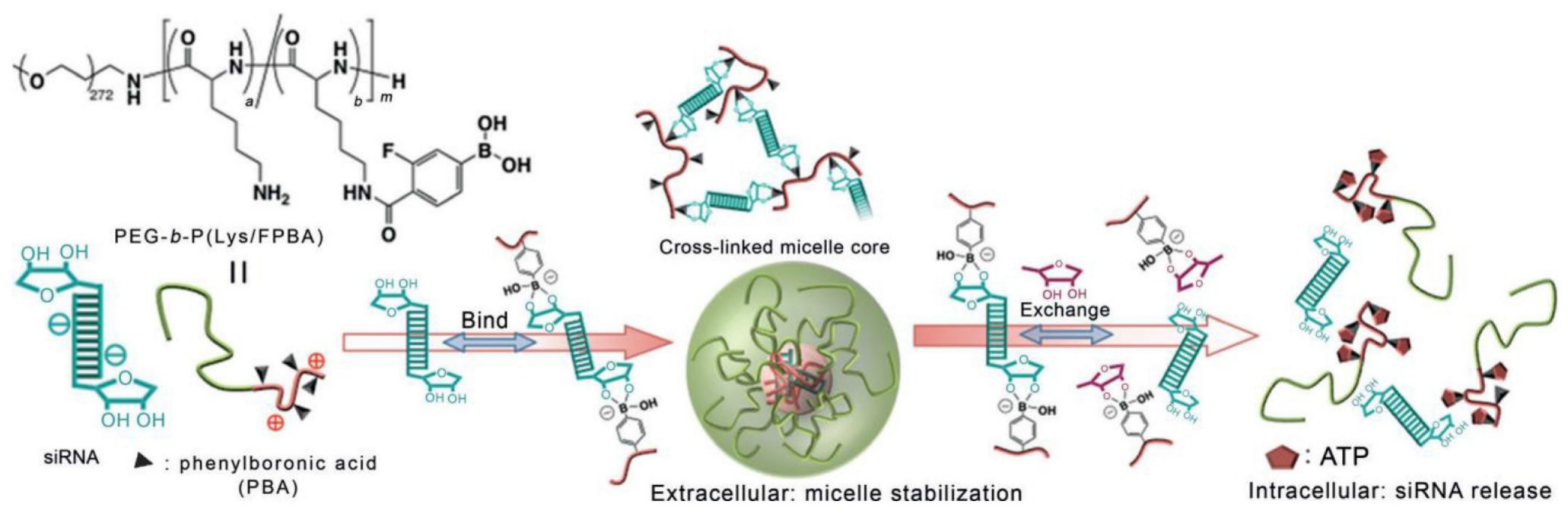
PBA-modified polymer cross-links with siRNA forming a stable micelle that releases siRNA in the presence of intracellular concentrations of ATP by Naito *et al.* Adapted with permission from 88, copyright 2012 Wiley.

On the other hand, the PBA derivatives can be incorporated into drug delivery systems as moieties designed for efficient and selective recognition of the cancer cells, enabling targeted action. In 2012, Kataoka and colleagues described the first such example of micelles built from the cationic polymer poly(ethylene glycol)-block-poly(L-lysine) whose lysine residues were further modified with a PBA derivative 88. The resulting polymer formed complexes with siRNA through the formation of a boronic ester bond with riboses of RNA. SiRNA cancer therapy has immense potential, but the lack of safe and effective delivery inhibits its clinical use, and this polymer offered a way to overcome these issues. Interestingly, the presence of ATP at intracellular concentrations triggered siRNA release (Figure 8), resulting in gene silencing of a well-known proto-oncogene in the human renal carcinoma cell line with minimal cytotoxicity.

**Figure 9:**
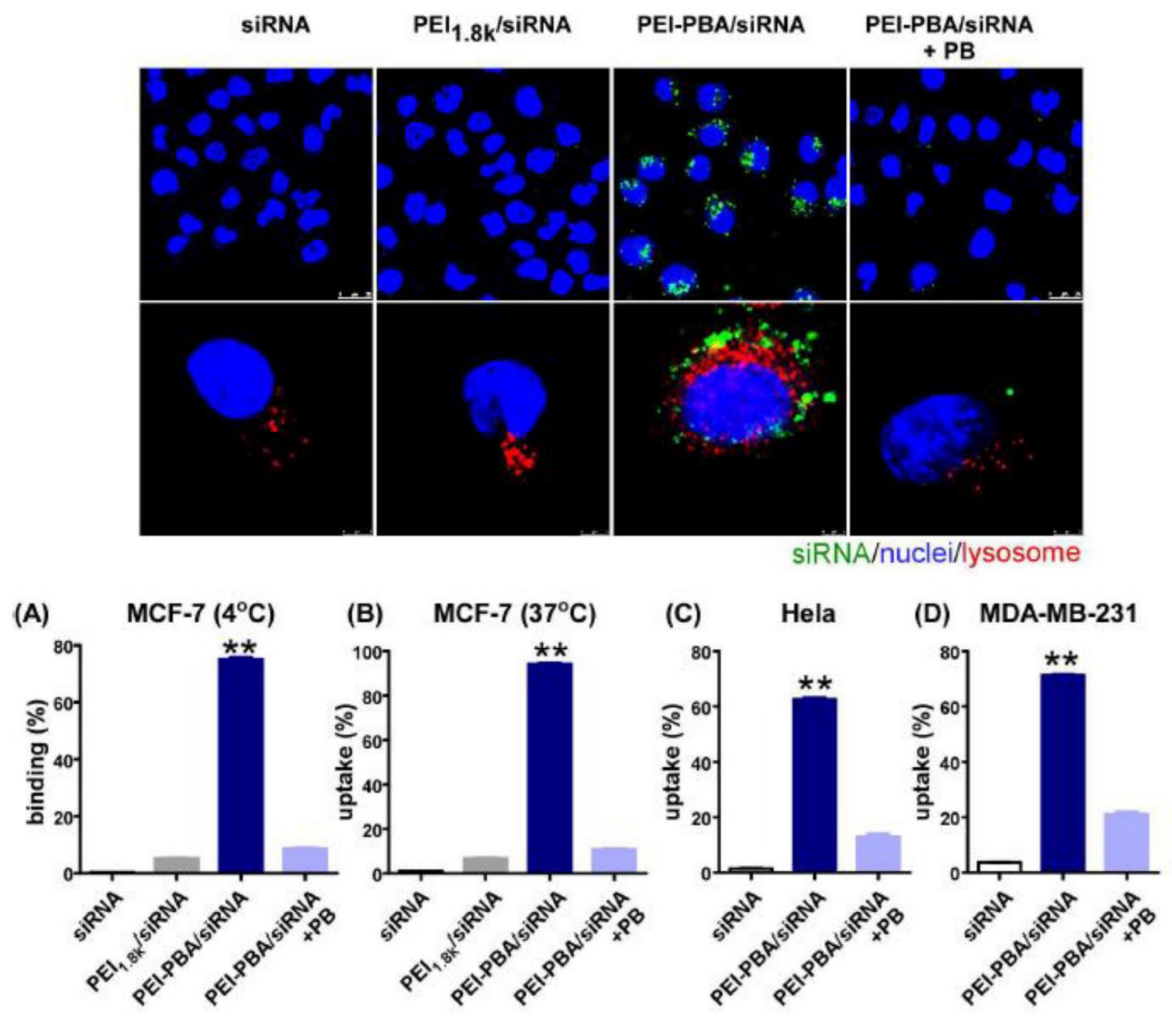
Cellular binding, uptake, and intracellular localization of siRNA in cancer cells. The uptake and binding of siRNA were quantified using flow cytometry. Adapted with permission from 89, copyright 2016 American Chemical Society.

A few years later, Ma *et al.* offered a similar PBA-based system for siRNA delivery based on PBA-grafted PEI 89. The nanocomplex formed between the polymer and siRNA had excellent biocompatibility, as well as serum stability, and was resistant to RNases. The PBA-SA interactions led to a dramatic increase in its uptake by cancer cells compared to free siRNA. Even more, the encapsulating polymer promoted the lysosome escape of siRNA which in turn decreased the expression of the target gene in cancer cells and induced tumor apoptosis and cell cycle arrest without causing any significant systemic toxicity or immunotoxicity. The microscopic images depicting cellular binding, uptake, and intracellular localization of siRNA in cancer cells, and the results of flow cytometry for binding and uptake are presented in Figure 9.

**Figure 10:**
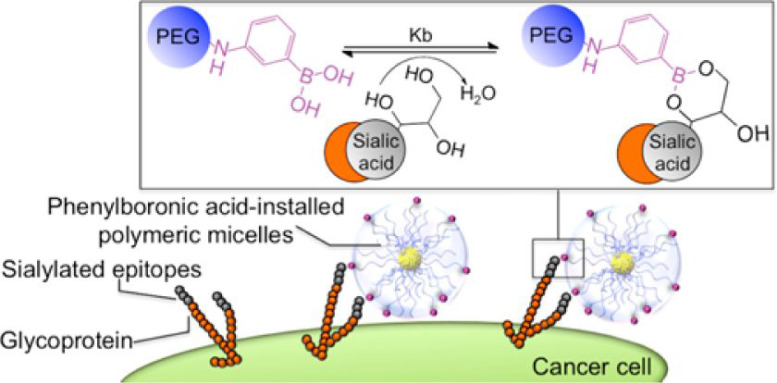
PBA decorated micelles loaded with oxaliplatin by Kataoka *et al.* and their interaction with cancer cells. Adapted with permission from 42, copyright 2013 American Chemical Society.

In 2013, the same group applied a similar approach to prepare micelles made of poly(ethylene glycol)-b-poly(L-glutamic acid), PEG-b-PLGA, with one end functionalized with PBA, as shown in Figure 10 42. These micelles were loaded with anticancer drug oxaliplatin and the obtained system demonstrated a higher affinity for SA compared to other common biologically important monosaccharides. Additionally, they showed increased cellular uptake in murine melanoma cells and enhanced antitumor effect compared to micelles lacking the PBA moiety. Furthermore, it was clearly demonstrated that this was a direct result of the interactions between PBA and SA on the surface of cancer cells which enhanced the micelles retention at the tumor giving rise to superior antitumor efficacy both *in vitro* as well as *in vivo*.

**Figure 11:**
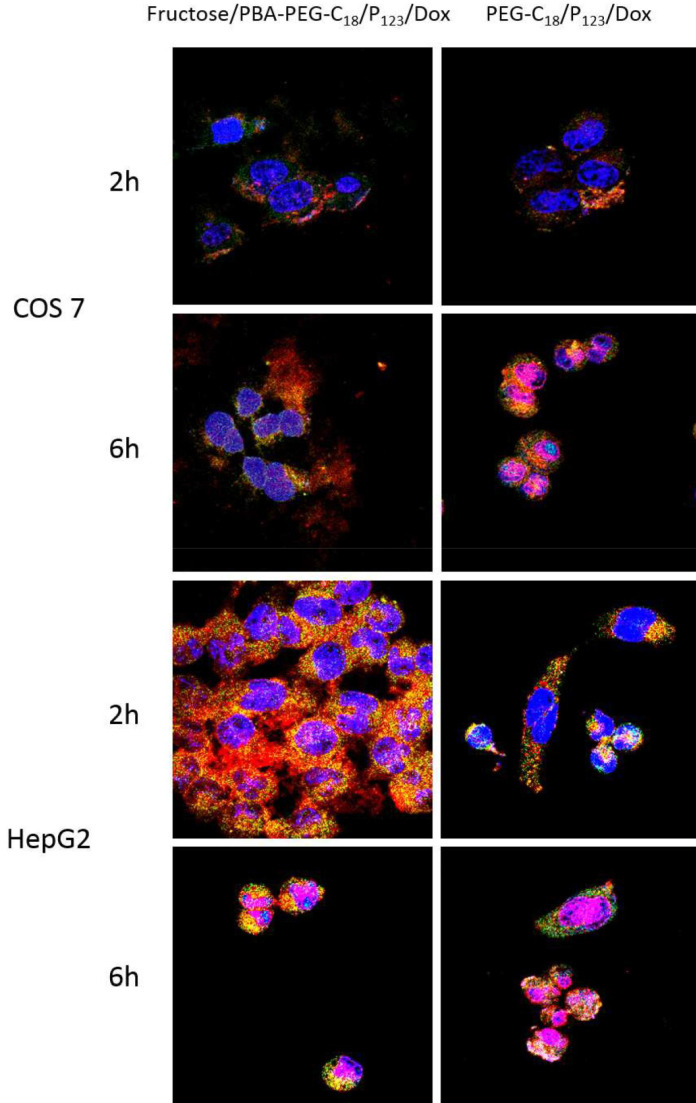
Confocal microscopic images of COS 7 cells at pH 7.4 and HepG2 cells at pH 6.5 showing subcellular distribution of fructose/PBA-PEG-C18/P123/Dox and mPEG-C18/P123/Dox (with stained nucleus and acidic compartments in live cells). Adapted with permission from 43, copyright 2016 American Chemical Society.

The development of pH-responsive nano-drug delivery systems incorporating PBA represented a notable advancement in the field. In 2016, Cheng, Li *et al.* took this concept further by introducing a simple yet efficient strategy: coating PBA-terminated polyethylene glycol monostearate and Pluronic P_123_ (PBA-PEG-C18) mixed micelles with fructose and loading them with DOX 43. At physiological pH, PBA-PEG-C18 preferentially binds to fructose over SA limiting its interactions with healthy cells and reducing the cytotoxicity of the DOX-loaded micelles toward them, while increasing their targeting ability for tumor cells. In contrast, at the lower pH of the tumor environment, the interactions with SA strengthen and over-compete with fructose, enabling targeted action. This design led to enhanced cellular uptake (Figure 11) by endocytosis and increased cytotoxicity of DOX-loaded micelles.

A similar strategy was employed by Kim *et al.*, who used crosslinked polyethylenimine (PEI) functionalized with PBA and PEI functionalized with galacturonic acid 90. The resulting cationic polymer gel exhibited stronger interactions with DNA than either component did by itself, suggesting that the polymer decomposition would lead to DNA release. Indeed, a lower pH, intracellular concentrations of ATP, or both triggered the disruption of the crosslinked polymer and the subsequent release of DNA from the previously stable polyplex. The delivery of DNA to the cell nucleus increases gene expression. This approach was used to deliver DNA as a tumor angiogenesis inhibitor, demonstrating that the system effectively targeted tumors and suppressed angiogenesis, highlighting its potential as a vector for therapeutic gene delivery.

In 2016, the Hu group presented an assay to measure SA expression on the surface of cancer cells 91. They employed PBA-biotin conjugates to selectively recognize the SAs and then attached to them gold NPs conjugated to streptavidin, known for its very high affinity towards biotin to enhance the inductively coupled plasma mass spectrometry (ICPMS) signal. This method demonstrated high selectivity, and competitive experiments provided accurate estimations of SA expression level on the cancer cell surface. Additionally, the assay successfully detected cancer cells, using SA as a biomarker, indicating its potential applicability for biological research and clinical diagnostics.

Xiao, Lu *et al.* previously described a nano-prodrug consisting of CPT conjugated with gemcitabine through a disulfide bond (CPT-SS-GEM), which showed synergistic antiproliferative efficacy in multiple cancer cell lines 92. However, the prodrug exhibited insufficient selectivity and tended to self-aggregate and precipitate at higher concentrations, limiting its potential medical application. To address these challenges, they developed a PEG polymer functionalized with PBA at one end and CPT at the other. The resulting polymer co-assembles with CPT-SS-GEM into stable rod-shaped nano-micelles with higher total drug concentration 93. Both the polymer and CPT-SS-GEM are redox-sensitive prodrugs, allowing them to degrade within the tumor microenvironment, enabling the delivery of two drugs with different mechanisms of action. *In vitro* experiments against a multidrug-resistant cancer cell line and murine breast cancer cells revealed enhanced cellular internalization and a strong synergistic anticancer effect.

In addition to drug delivery systems, Huang and collaborators pioneered the development of PBA-functionalized co-delivery micelles aiming to overcome multidrug resistance in cancer treatment 94. The micelles consisted of PBA-functionalized PEG-polyglutamic acid polymer with disulfide-bonded CPT and loaded with DOX. The drug ratio resulting in maximal synergistic effect was determined. Additionally, it was shown that the micelles efficiently released the drug in lysosomal conditions, but not in plasma. Also, they demonstrated higher endocytosis and lower drug efflux rates than conventional drugs. Notably, the study suggests that these micelles may act by downregulating specific factors, further emphasizing their potential in combating multidrug resistance. This work represents a significant advancement in cancer therapy, offering a promising strategy to improve drug delivery and efficacy in multidrug-resistant tumors.

In 2022, Zhang introduced linear and star PEG-poly(l-boronophenylalanine) copolymers that self-assembled into micellar NPs 95. The NPs co-encapsulated antitumor drugs curcumin and sorafenib tosylate and exhibited dual release as a response to H_2_O_2_ and acidic environment resulting in greatly enhanced anticancer effect. The relatively easy synthesis of the NPs, robust drug loading and responsive drug release make this system highly interesting for further development of smart cancer nanomedicine.

Li 96 and Wang 97 highlight the potential of PBA-based functional materials in fluorescence imaging and tumor therapy. Wang prepared unimolecular polymer nanomaterials consisting of poly(tert-butyl acrylate) whose arms were functionalized with PBA, DOX and PEG. The incorporation of PBA groups provided specific tumor targeting and high accumulation in tumors, while the presence of a pH-sensitive acylhydrazone linkage between the polymer chain and DOX ensured rapid DOX release in tumor tissues, thus promoting the therapeutic efficacy and enhancing the overall anti-cancer effect.

### 6.3. Other types of PBA-decorated nanosystems for antitumor therapy

Although most PBA-based DDS used PEG and PEI as drug carriers due to their biocompatibility and the resulting medical applicability, several alternatives emerged.

One of the earliest examples of diagnostic applications was PBA-conjugated quantum dot probes, developed by Duan *et al.* 98, for effective imaging and tracking of SAs. The study employed single-particle imaging techniques to quantitatively analyze the PBA-SA interactions and demonstrate the average number of PBA groups per QD and their effective targeting capabilities. These findings established PBA-conjugated QD probes as valuable tools for studying glycan dynamics and SA-related biological processes related to sialic acids, with implications for understanding glycosylation in various diseases.

**Figure 12:**
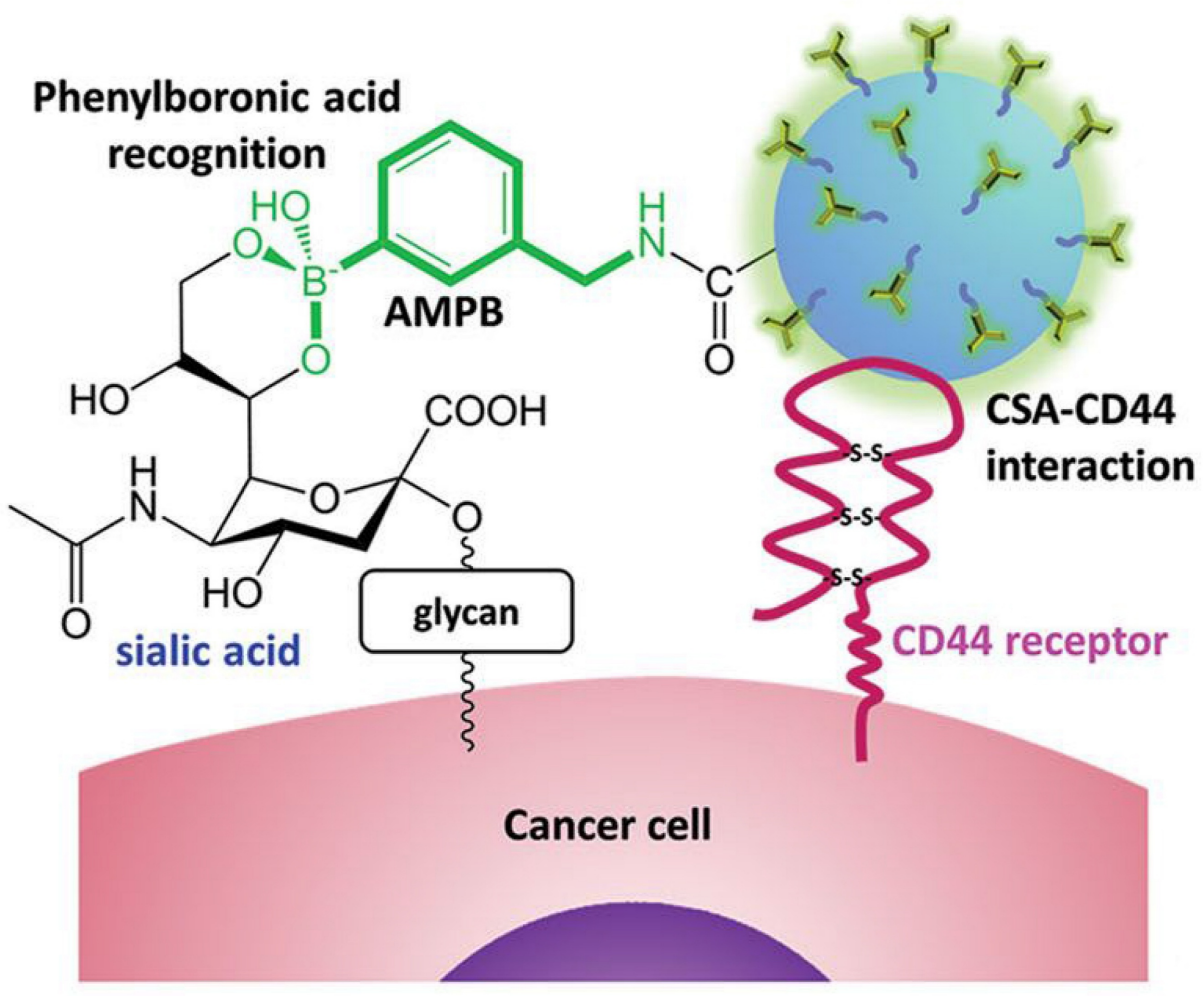
Tumor targeting of paclitaxel-loaded NPs by Cho, Kim, *et al.* Adapted with permission from 99, copyright 2015 Wiley.

Several years later, Cho, Kim, and coworkers developed NPs composed of biocompatible chondroitin sulfate A (CSA), known for binding to the CD44 receptor expressed in cancer cells, conjugated with Cy5.5 fluorescent dye and deoxycholic acid functionalized with PBA *via* an amide bond (Figure 12) 99. The NPs were loaded with doxorubicin (DOX), and a decrease in pH was shown to enhance DOX release. Additionally, the cellular uptake and penetration into human lung adenocarcinoma cells were improved compared to non-PBA-functionalized NPs both *in vitro* and *in vivo*. Dual tumor targeting achieved through PBA-SA and CSA-CD44 interactions enhanced cellular uptake leading to suppression of the tumor growth by increasing apoptosis, while maintaining low cytotoxicity to healthy cells.

An interesting advancement was made by Cui, Li, and coworkers who presented an improved fluorescent conjugated polymer modified with PBA for targeted cancer cell imaging 100. They used SAs as templates during the reprecipitation part of poly(fluorene-alt-benzothiadiazole) NP preparation which were later removed by adjusting the pH. The resulting NPs exhibited improved properties compared to the unmodified NPs, including strong fluorescence and ability to discriminate between cancer cells and normal cells through binding to SAs overexpressed on the surface of cancer cells resulting in their selective staining. The study also demonstrated low cytotoxicity of the NPs, indicating their biocompatibility and potential for use as fluorescent probes for live-cell imaging.

Building upon the exploration of PBA and its derivatives in drug delivery systems, Kang *et al.* 101 presented a poly(methyl vinyl ether-*alt*-maleic acid) conjugated with PBA loaded with DOX. The resulting nanocomplex exhibited enhanced anti-tumor activity through tumor-specific targeting in different cancer cells. By targeting the overexpressed Ss, the nanocomplexes enabled precise doxorubicin delivery.

Jung *et al.* 102 also focused on PBA-functionalized copolypeptides for enhanced anticancer effects through responsive drug release. They prepared PBA-grafted poly(methyl vinyl ether-*alt*-maleic acid) with a goal to improve the solubility issues of curcumin as a drug. The coordination bonding between 1,3-dicarbonyl compounds and PBA enabled high drug loading capacity of the polymer and pH-responsive drug release mechanism. The platform exhibited promising therapeutic efficacy, showing both chemotherapeutic and anticancer effects.

Finally, in their recent study, Demirbuken *et al.* 103 introduced artificial motors as a novel approach to drug delivery. Inspired by natural motors, these artificial counterparts have attracted significant interest for their remarkable capabilities and potential applications. Among them, miniaturized nano/micromotors have emerged as versatile tools for (bio)sensing, cargo transport, and drug delivery. The study focused on PTX-conjugated robots featuring a poly(3-aminophenylboronic acid) outer layer, a platinum (Pt)-nickel (Ni) segment, and a Pt catalytic inner layer. These self-functionalized motors exhibited efficient autonomous movement and controlled drug release, particularly under near-infrared (NIR) irradiation. Fabricated through electrochemical methods, the motors demonstrated targeted drug delivery to human breast cancer cells, showcasing pH-dependent behavior. The study highlighted their promising viability and drug release efficiency, underscoring their potential for biomedical applications.

## 7. Conclusions and future prospects

Cancer remains one of the leading causes of death globally, with nearly 10 million deaths and around 200 million new cases reported annually, according to the World Health Organization (WHO) 104,105. Conventional treatments, including surgical procedures, radiation therapy, and pharmacological approaches such as chemotherapy, hormone therapy, and targeted biological therapies, are widely employed. However, these methods are often accompanied by significant and severe side effects. This is primarily due to the lack of specificity of many drugs, which, while targeting cancer cells, also inevitably impact healthy cells as they circulate through the body. Consequently, recovery can become prolonged, more challenging, and unpredictable.

Therefore, it is not surprising that the search for targeted diagnostic tools and therapies remains a key focus of cancer research, with PBA-based systems emerging as a highly promising foundation for further developments. PBA derivatives and PBA-functionalized drug delivery systems have shown potential for various biomedical and therapeutic applications 106, primarily due to their unique ability to selectively bind to SA overexpressed on cancer cells surfaces. This specificity also makes the PBA moiety a perfect platform for the development of targeted cancer therapies and diagnostic tools. Over time, PBA-based systems for diagnostics and therapies have evolved from simple derivatives to complex nanomaterials exhibiting improved therapeutic efficacy and reduced off-target effects. This evolution is clearly visible in Figure 2 and emphasizes their important advantages compared to conventional anticancer drugs: targeted drug delivery, increased bioavailability, enhanced therapeutic efficacy, and reduced systemic toxicity. Even more, numerous PBA-based formulations have shown significant potential for use in imaging techniques. Through diverse and innovative approaches, in the last several years, a wide array of very versatile materials and technologies that hold promise in the fight against cancer was developed, translating nature-inspired research into innovative therapeutic strategies with the potential for significant impact in clinical practice. Moreover, although the effect of PBA-derivatives has so far been studied on only several enzymes, the recent emergence of new tools for protein design and structure elucidation recognized with the 2024 Nobel Prize in Chemistry will surely propel research and enable design of specific enzyme-targeting strategies.

As discussed in previous sections, preclinical and clinical trials have offered compelling evidence about the efficiency of PBA-based anticancer agents. However, their primary limitation is their potential toxicity to healthy cells. Since SA is present on both healthy and cancer cell surfaces, PBA-based agents can bind to both. Nevertheless, the binding affinity of PBA towards SA is pH dependent and somewhat lower at the pH of healthy cells than in a more acidic environment of cancer cells. Combined with significantly lower SA expression on healthy cells, this leads to selective targeting of cancer cells and reduced, although not entirely eliminated, impact on non-cancerous tissues. Future research and drug development efforts will likely focus on further increasing this discrepancy and selectivity in order to maximize the therapeutic effect of PBA-based systems while minimizing off-target effects.

The highly promising results of preclinical *in vitro* and *in vivo* studies justify the strong interest of the scientific community and lay the groundwork for further development. As this young field continues to rapidly evolve, optimizations of the existing PBA-based systems, as well as development of new strategies to harness the unique properties of PBA, are expected to enhance treatment efficacy and improve outcomes for cancer patients. Modern medicine is shifting towards precision and personalized approaches, so given the exceptional potential of PBA-based drug delivery systems to revolutionize cancer diagnosis and treatment, research is expected to continue expanding through innovations, eventually reaching clinical use. Such advancements could provide groundbreaking personalized cancer therapies, state-of-the-art diagnostic tools, and multifunctional drug delivery systems with profound implications for clinical practices.
